# Genotype and injury severity modulate the effects of traumatic brain injury on sleep in *Drosophila*

**DOI:** 10.17912/micropub.biology.002034

**Published:** 2026-03-16

**Authors:** Leah Pisano, Michael P. Shahandeh

**Affiliations:** 1 Lynbrook Senior High School, Lynbrook, New York, United States of America; 2 Department of Biology, Hofstra University, Hempstead, New York, United States of America

## Abstract

Traumatic brain injuries (TBIs) have many negative impacts in humans (i.e. lifespan, cognitive function, sleep). Recently, *Drosophila* emerged as a model for studying these impacts in a controlled environment. Previous studies have described effects of TBIs on sleep in *Drosophila* , but how these effects are modulated by injury severity or genetic background has not been explicitly investigated. Here, we exposed male *Drosophila melanogaster* from two genetic backgrounds to two TBI severity treatments and measured effects on sleep. We document significantly increasing effects with injury severity in both genetic backgrounds and describe differences between genotypes, demonstrating a genetic basis to susceptibility.

**Figure 1. Genotype and severity of traumatic brain injury (TBI) effects on sleep in Drosophila:**
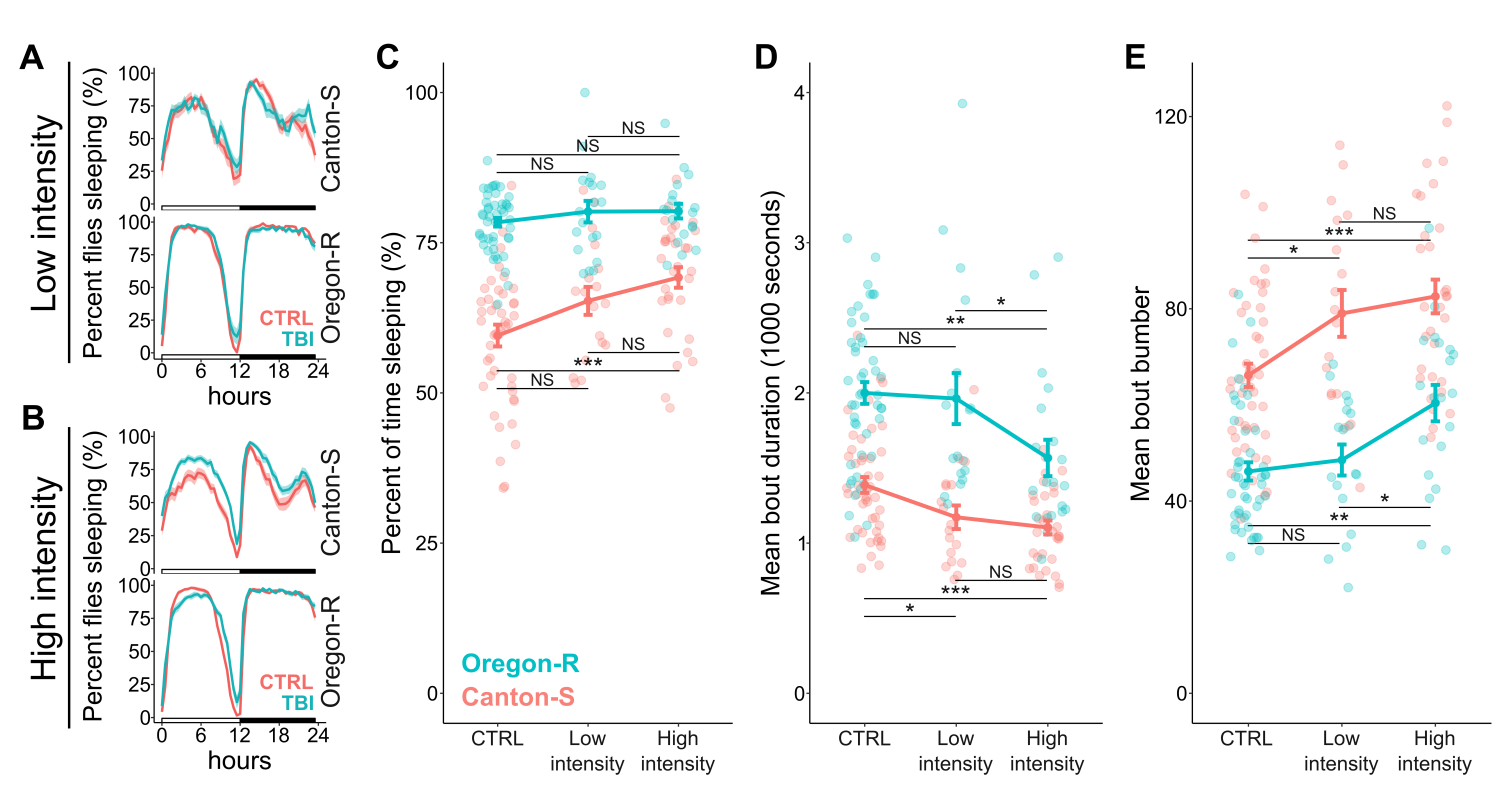
**A.** The total proportion of flies asleep under control conditions (no TBI, red) and low intensity TBI (blue) conditions across a 24-hour period. **B.** The total proportion of flies asleep under control conditions and high intensity TBI conditions across a 24-hour period. For **A** and **B** , the data shown represent the average for all flies across a 4-day sampling period. **C.** The percent of time individual flies spent sleeping across a 4-day sampling period under control, low intensity TBI, and high intensity TBI conditions. **D.** The mean sleep bout duration in seconds for individual flies of the same conditions. **E.** The mean number of sleep bouts per day for individual flies of the same conditions. For **C** - **E** , Canton-S fly data is depicted in red and Oregon-R data is depicted in blue. Bold points connected by lines depict mean values and error bars depict the standard error of the mean. Asterisks denote significance detected by Wilcoxon rank-sum tests followed by post-hoc Bonferroni correction (NS = not significant, * = p < 0.05, and *** = p < 0.001). Sample sizes: Canton-S control flies (45), Oregon-R control flies (46), Canton-S low intensity TBI flies (18), Oregon-R low intensity TBI flies (18), Canton-S high intensity TBI flies (31), and Oregon-R high intensity TBI flies (20).

## Description

Traumatic brain injuries (TBIs) constitute a major public health concern for humans that are associated with many conditions, including sleep disorders (Obasa et al., 2024). However, studying these effects in humans is difficult, as it can only be done in a clinical setting where the severity of injury is difficult to control, as are patient demographics and environment. Additionally, human studies are limited in their ability to investigate the molecular determinants of TBI effects. Recently, *Drosophila* has arisen as a model for the study of TBIs and their effects in a controlled laboratory environment (reviewed in: Shah et al., 2019). In flies, TBIs have pervasive negative effects on behavior, survival, and cause neuronal phenotypes similar to what has been described in mammals (Barekat et al., 2016; Katzenberger et al., 2013; Saikumar et al., 2020). Due to *Drosophila* ’s relatively short lifespan, these effects can be studied longitudinally to understand their molecular underpinnings, detect secondary injury cascades, and identify optimal therapeutic windows in a controlled laboratory environment (Buhlman et al., 2021). Previous studies have shown an effect of TBIs on sleep in *Drosophila* , with flies experiencing more disrupted sleep with increasing TBI severity (Barekat et al., 2016; van Alphen et al., 2022). However, these studies used a single *Drosophila* genotype and repeated TBI induction (5 – 10 times) to detect these effects. Given our understanding that genetic factors can influence TBI outcomes in mammals (Jordan, 2007), it is important to understand if and how different genotypes will respond to TBI. Additionally, it remains unknown if and how lower-intensity TBIs influence sleep. &nbsp;&nbsp;&nbsp;&nbsp;&nbsp;&nbsp;&nbsp;&nbsp;&nbsp;&nbsp;

To fill this gap in knowledge, we use two isogenic inbred laboratory strains of *Drosophila melanogaster* , representing independently sourced genetic backgrounds (Canton-S and Oregon-R). We used a previously validated ‘HIT’ device (Katzenberger et al., 2013) to subject these strains to both ‘low intensity TBI’ (1 strike) and ‘high intensity TBI’ (3 strike) conditions (see ‘Methods’). We then monitored their daily activity to test the following hypotheses regarding the effects of brain injury on sleep: (1) Flies will display increasingly impaired sleep phenotypes with increasing TBI severity; and (2) certain genotypes will be more susceptible to the effects of TBI, displaying sleep impairment even when TBIs are lower-intensity.

When comparing daily sleep profiles between genotypes, there were obvious genotype-specific differences in average sleep pattern ( Figure 1A- B). We therefore first compared control flies with no TBI between genotypes and found that Canton-S spends significantly less time sleeping, displays, on average, shorter sleep bouts, and has more sleep bouts per day than Oregon-R (p = 4.9e-13, p = 1.5e-08, and p = 1.5e-08 respectively; Wilcoxon test). Thus, there are already natural differences in sleep phenotypes, resulting from genetic differences between these strains, that create the potential for different interactions between genotype and TBI treatment. To investigate these potential interactions, we performed 2-way ANOVA to test both for an individual effect of treatment, and for a potential interaction between treatment and genotype.

For percent time sleeping, we detect both a significant effect of treatment and a significant interaction between treatment and genotype (Table 1), indicating that Canton-S and Oregon-R display different effects of TBI severity on overall sleep. This is driven by no significant change on overall sleep with increasing TBI severity for Oregon-R, but a significant increase in overall sleep for Canton-S when comparing the high-intensity TBI treatment to low intensity TBI and controls ( Figure 1B ). For mean sleep bout duration, we detect a significant effect of treatment, but no significant interaction between treatment and genotype (Table 2). Nonetheless, when we compare between treatments within each strain, we find that Canton-S displays reduced sleep bout duration already under low intensity TBI conditions, with no change from low to high intensity TBI, while Oregon-R only displays a significant decrease in sleep bout duration under high intensity TBI conditions ( Figure 1C ). We observe the same pattern for mean bout number—2-way ANOVA detects an effect of treatment, but no interaction between genotype and treatment (Table 3). However, comparison within genotypes indicates that Canton-S displays increased bout number under low intensity TBI conditions, with no change from low to high intensity, while Oregon-R flies do not display a significant increase in bout number until they receive high intensity TBIs ( Figure 1D ).

Overall, our results confirm previous findings that flies that received TBIs showed disrupted sleep (Barekat et al., 2016; van Alphen et al., 2022). However, we additionally show that this effect was sometimes dependent both on genetic background and on TBI severity. In both genotypes, flies display increased sleep fragmentation, with shorter and more frequent bouts of sleeps observed under TBI conditions relative to controls, however these effects appeared under low intensity TBI conditions for Canton-S, but only under high-intensity TBI conditions for Oregon-R. Interestingly, we only observed changes in overall sleep for one genotype, Canton-S, demonstrating genetic differences in susceptibility to the effects of TBIs on this sleep metric. While these results demonstrate that injury severity and genotype modulate the effects of TBIs on sleep in *Drosophila* , they also highlight areas for future investigation. For instance, here we only used young males, as is standard with *Drosophila* activity monitoring (Pfeiffenberger et al., 2010). However, there are likely sex and age interactions with TBI effects (Ye et al., 2023). With the present study, we are unable to extend our results to females or flies of older age, thus these variables warrant further investigation. Likewise, here we have chosen to average sleep across a four-day observation period following 48 h of recovery from TBI administration to specifically examine persistent effects of TBIs on sleep. However, it is possible that this approach misses or obscures transient effects of TBIs on sleep that resolve early after injury. Future investigations of potential transient effects of injury severity and genotype on sleep immediately following TBI occurrence also warrant further investigation.

## Methods

*Drosophila strains and maintenance*

We maintained two wild-type laboratory strains (Table 4) of *D. melanogaster* on non-overlapping alternating 2-week cycles at 25°C and ~50% relative humidity under a 12 h light/dark cycle on a cornmeal-molasses-yeast media (0.9% agar, 2.6% yeast, 6.25% cornmeal, 6.25% molasses). 1–3-day old males were collected in groups of 10 under light CO _2_ anesthesia and allowed to recover for 48 hours prior to the administration of TBIs.

*Traumatic brain injuries*

We replicated a previously published and validated machine for administering TBIs to flies (Katzenberger et al., 2013), where flies are slammed at high velocity against the side of a container via a metal spring, inducing head trauma. In brief, 3-5-day old flies were transferred without anesthesia to a 14 mL test tube (USA Scientific #5618-7262) and inserted into a ring at the end of a horizontal metal spring mounted to a wooden board. The metal spring was raised to a 90-degree angle and released. One strike was used for our low intensity TBI treatment, and 3 strikes, separated by 5 minutes of recovery, for our high intensity TBI treatment. Following release, we regularly observed temporary loss of consciousness among our flies. Control flies were transferred to a 14 mL test tube and inserted into the device, but no strikes were administered.

*Activity monitoring*

To ensure long-term survival of the flies used to measure sleep, after administration of TBIs, flies were allowed to recover for 48 hours before activity monitoring. After recovery from TBI induction, 5-7-day old males were transferred to the *Drosophila* Activity Monitor (DAM) system to record their daily activity rhythms (Pfeiffenberger et al., 2010). In brief, under quick CO _2_ anesthesia, flies were transferred to glass capillary tubes containing a 2% agar (BD Difco Agar #214010) 5% sucrose (ThermoScientific #036508.A1) solution on one end and sealed with a cotton plug at the other. 32 tubes are housed within a single monitor which uses an infrared beam to count the number of times per minute each fly crosses the midline as an approximation of activity. Monitors were stored in incubators at 25°C and ~50% relative humidity under a 12 h light/dark cycle for four days. The resulting data were analyzed and visualized using the Rethomics package in R (Geissmann et al., 2019). Sleep bouts were defined as any period of inactivity that extended 5 minutes or beyond (Beckwith & French, 2019).

Statistical analysis

To ensure consistency across experiments, we performed two technical replicates each for our low intensity and high intensity TBI treatments, always with their own controls. Following data collection, we compared control flies across these replicates, and found no significant differences between uninjured flies for percent of time sleeping (p =0.77234 for Canton-S and p = 0.37217 for Oregon-R), sleep bout duration (p = 1 for both), or mean number of sleep bouts per day (p = 1 for both) using a Wilcoxon test with post-hoc correction for multiple comparisons (although, no p-values were significant prior to correction). Thus, with no evidence of significant variation between our replicates, we pooled the technical replicates across both treatments to directly test for differences between them. On the pooled data, we performed 2-way ANOVA for percent of time sleeping, sleep bout duration, and mean number of sleep bouts per day to investigate an effect of genotype (Canton-S or Oregon-R), treatment (control flies, low intensity, and high intensity TBI), and a potential interaction between the two. Results are presented in table format below. Within each strain, we compared the percent of time flies spent sleeping, mean sleep bout duration, and mean sleep bouts per day between all treatments using Wilcoxon rank-sum tests with post-hoc correction for multiple comparisons (Holm, 1979). The results of these tests are depicted on Figure 1. &nbsp;

Table 1. Results from 2-way ANOVA for percent of time sleeping.

**Table:** 

	Df	Sum Sq	Mean Sq	F value	Pr(>F)
Genotype	1	1.0695	1.0695	133.387	< &nbsp;2e-16***
Treatment	2	0.1352	0.0676	8.432	0.00032***
Genotype*Treatment	2	0.0499	0.025	3.112	0.04701*
Residuals	174	1.3952	0.008		

&nbsp;

Table 2. Results from 2-way ANOVA for mean sleep bout duration.

**Table:** 

	Df	Sum Sq	Mean Sq	F value	Pr(>F)
Genotype	1	17949241	17949241	88.949	< &nbsp;2e-16***
Treatment	2	3792578	1896289	9.397	0.000134***
Genotype*Treatment	2	555158	277579	1.376	0.255463
Residuals	174	34708453	201793		

&nbsp;

Table 3. Results from 2-way ANOVA for mean sleep bout number per day.

**Table:** 

	Df	Sum Sq	Mean Sq	F value	Pr(>F)
Genotype	1	25509	25509	94.442	<&nbsp; 2e-16***
Treatment	2	7650	3825	14.16	2.03E-06***
Genotype*Treatment	2	717	359	1.328	0.268
Residuals	174	46458	270		

## Reagents

Table 4. *Drosophila* reagents.

**Table:** 

Strain name	Genotype	Bloomington *Drosophila* stock center ID
Canton-S	*wild-type*	RRID:BDSC_64349
Oregon-R	*wild-type*	RRID:BDSC_2376
